# Biocatalytic Desulfurization Capabilities of a Mixed Culture during Non-Destructive Utilization of Recalcitrant Organosulfur Compounds

**DOI:** 10.3389/fmicb.2016.00266

**Published:** 2016-03-03

**Authors:** Wael Ismail, Wael S. El-Sayed, Abdul Salam Abdul Raheem, Magdy E. Mohamed, Ashraf M. El Nayal

**Affiliations:** ^1^Environmental Biotechnology Program, Life Sciences Department, College of Graduate Studies, Arabian Gulf UniversityManama, Bahrain; ^2^Biology Department, Faculty of Science, Taibah UniversityAl-Madinah Al-Monawarah, Saudi Arabia; ^3^Botany and Microbiology Department, Faculty of Science, Cairo UniversityGiza, Egypt

**Keywords:** mixed cultures, biodesulfurization, dibenzothiophene, *stenotrophomonas*, 4S pathway

## Abstract

We investigated the biodesulfurization potential of a mixed culture AK6 enriched from petroleum hydrocarbons-polluted soil with dibenzothiophene (DBT) as a sulfur source. In addition to DBT, AK6 utilized the following compounds as sulfur sources: 4-methyldibenzothiophene (4-MDBT), benzothiophene (BT), and 4,6- dimethyldibenzothiophene (4,6-DM-DBT). None of these compounds supported the growth of AK6 as the sole carbon and sulfur source. AK6 could not grow on dibenzylsulfide (DBS) as a sulfur source. The AK6 community structure changed according to the provided sulfur source. The major DGGE bands represented members of the genera *Sphingobacterium, Klebsiella, Pseudomonas, Stenotrophomonas, Arthrobacter, Mycobacterium*, and *Rhodococcus*. *Sphingobacterium* sp. and *Pseudomonas* sp. were abundant across all cultures utilizing any of the tested thiophenic S-compounds. *Mycobacterium/Rhodococcus* spp. were restricted to the 4-MDBT culture. The 4-MDBT culture had the highest species richness and diversity. Biodesulfurization of DBT by resting cells of AK6 produced 2-hydroxybiphenyl (2-HBP) in addition to trace amounts of phenylacetate. AK6 transformed DBT to 2-hydroxybiphenyl with a specific activity of 9 ± 0.6 μM 2-HBP g dry cell weight^−1^ h^−1^. PCR confirmed the presence in the AK6 community of the sulfur-specific (4S) pathway genes *dszB* and *dszC*. Mixed cultures hold a better potential than axenic ones for the development of a biodesulfurization technology.

## Introduction

The increasing global human population accompanied by extensive fossil energy consumption has posed serious threats to the environment and human health (Maass et al., 2015). Organosulfur (thiophenes) compounds found in crude oil and diesel are of particular concern due to their hazardous impact on human health and the ecosystem (Kilbane, 2006; Morales et al., 2010). Moreover, the sulfur oxide gases resulting from fuel combustion are a major cause of acid rain. Governments and environmental organizations worldwide have recognized the problem and implemented strict regulations and legislations that limit the amount of sulfur in transportation fuels.

Hydrodesulfurization (HDS) is commonly applied by oil refineries to reduce sulfur content in refined products (Konishi et al., 2000). Nonetheless, HDS has many disadvantages. It is cost-intensive, environmentally polluting, and not sufficiently efficient. Consequently, there has been an increasing interest in the development of alternative desulfurization technologies that circumvent the drawbacks associated with the conventional HDS (Ohshiro and Izumi, 1999). The petroleum refineries are facing the problem that crude oil feeds are becoming heavier with high sulfur content, which means high sulfur levels in both straight-run and secondary processed diesel oil (Bhatia and Sharma, 2006). This will make the HDS process more economically and technically challenging, particularly with the stringent environmental legislations that tend to limit sulfur content of transportation fuels to less than 10 ppm (Monot and Warzywoda, 2008).

Non-destructive (sulfur-specific) microbial desulfurization or biodesulfurization (BDS) has been proposed as an alternative or complementary technology. BDS exploits the ability of dedicated microorganisms to remove sulfur from many organosulfur compounds that are commonly found in crude oil and diesel. As compared to physicochemical techniques like HDS, biocatalytic processes are environmentally friendly, cost-effective, specific, and more efficient (Kilbane, 2006). During the past two decades, many microorganisms have been isolated and characterized based on their unique ability to specifically remove the sulfur atom from organosulfur substrates without breaking the carbon skeleton. This desulfurization mechanism preserves the calorific value of the treated fuel.

**Figure 1:**
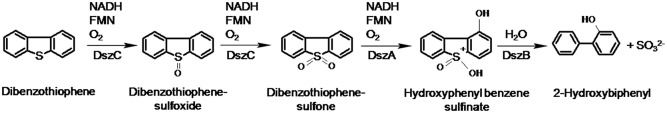
**The 4S pathway of non-destructive biodesulfurization of dibenzothiophene (Gallagher et al., 1993)**.

The most common biodesulfurization pathway reported to date is the 4S pathway discovered initially in *Rhodococcus erythropolis* IGTS8 (Gallagher et al., 1993; Figure 1). The 4S pathway is well-characterized at the biochemical and molecular levels. It proceeds via two cytoplasmic monooxygenases (DszC, DszA) supported by a flavin reductase (DszD) and a desulfinase (DszB). DBT monooxygenase (DszC) catalyzes the sequential conversion of DBT to DBT sulfoxide (DBTO) and DBT-sulfone (DBTO_2_). DBTO_2_ monooxygenase (DszA) catalyzes the oxidative C-S bond cleavage producing 2-(2′-hydroxybiphenyl) benzene sulfinate (HBPS). DszB, an aromatic sulfinic acid hydrolase, affects a nucleophilic attack of a base-activated water molecule on the sulfinate sulfur to produce 2-hydroxybiphenyl (2-HBP) as a dead-end product and sulfite as a bioavailable sulfur for microbial growth. DszD delivers the reducing equivalents (FMNH_2_) needed for the functionality of DszC and DszA. The oxygen atom incorporated at each step of the pathway is derived from atmospheric oxygen.

The genes involved in DBT desulfurization (*dszA, dszB, dszC*) are organized as one operon (*dsz* operon) and transcribed in the same direction under the control of a single promoter. The three genes are clustered on a 120-kb linear plasmid of the *R. erythropolis* IGTS8 strain. A fourth gene, *dszD*, that encodes a flavine reductase is located on the chromosome.

Despite the progress that has been achieved during the last two decades, biodesulfurization has not been applied on a commercial scale yet. This is attributed to many issues related to the stability and catalytic efficiency of the microbial biocatalyst in addition to other technical problems (Monot and Warzywoda, 2008). The majority of the research conducted on microbial desulfurization has adopted axenic cultures of selected microorganisms. However, it is worth investigating biodesulfurization capabilities of microbial consortia to benefit from the cooperative or synergistic microbe-microbe interactions (McGenity et al., 2012; Mikesková et al., 2012). Recently, engineered synthetic bacterial consortia have shown enhanced desulfurization and revalorization of oil sulfur compounds (Martínez et al., 2016).

The aim of this study was to enrich a mixed culture from soil polluted with petroleum hydrocarbons and to study its biocatalytic desulfurization potential using various organosulfur compounds as a sulfur source. Furthermore, we investigated the dynamics of the microbial consortium when challenged with different sulfur sources.

## Materials and methods

### Chemicals

Organosulfur compounds were purchased from Fluka (Switzerland), Acros (USA), and Sigma-Aldrich (USA). Other chemicals and culture media were from Difco (France), Fluka (Switzerland), Sigma (USA), Qiagen (Germany), and Promega (USA). Deionized water was used to prepare all media and solutions.

### Soil samples and bacteria

Soil samples contaminated with used motor lubricating oil, diesel, benzene, and grease were collected (top surface layer, 10 cm in depth) from the neighborhood of mechanic workshops in Fahaheel district, Kuwait. The AK6 bacterial consortium was enriched from the contaminated soil. A reference biodesulfurization strain, *R. erythropolis* IGTS8, was obtained from The American Type Culture Collection (ATTC 53968, USA).

### Culture media and growth conditions

Commercially available Lauria-Bertani (LB) agar and broth media were prepared according to the instructions of the supplier. Sulfur-free chemically defined medium (CDM) had the following composition (per litter): KH_2_PO_4_ 1.08 g; K_2_HPO_4_, 5.6 g; NH_4_Cl, 0.54 g; MgCl_2_.6H_2_O, 0.2 g; CaCl_2_.2H_2_O, 0.044 g; FeCl_2_.4H_2_O, 1.5 mg, vitamins (cyanocobalamin 0.2 mg, pyridoxamine-HCl 0.6 mg, thiamin-HCl 0.4 mg, nicotinic acid 0.4 mg, p-aminobenzoate 0.32 mg, biotin 0.04 mg, Ca-pantothenate 0.4 mg), and trace elements (ZnCL_2_.7H_2_O 70 μg, MnCl_2_.4H_2_O 100 μg, CuCl_2_ 20 μg, CoCl_2_.6H_2_O 200 μg, Na_2_MoO_4_.2H_2_O 40 μg, NiCl_2_.6H_2_O 20 μg, H_3_BO_3_ 20 μg). Routinely, the carbon source was glucose (10 mM) and the sulfur source was either MgSO_4_.7H_2_O (1 mM) or an organosulfur compound (0.1 mM). The tested organosulfur substrates were dibenzothiophene (DBT), benzothiophene (BT), 4-methyldibenzothiophene (4-MDBT), 4,6-dimethyldibenzothiophene (4,6-DM-DBT), and dibenzylsulfide (DBS). All organosulfur compounds were added to the CDM from 100 mM ethanol stocks except 4,6-DM-DBT which was prepared in acetone. The final concentration of either ethanol or acetone in the culture media was 0.1% (vol/vol). MgSO_4_ was replaced by MgCl_2_.6H_2_O when organosulfur compounds were used either as the sole sulfur source or as the sole sulfur and carbon source (in this case glucose was omitted). All liquid cultures were incubated in an orbital shaker (180 rpm) at 30°C. All cultures on solid media were incubated at 30°C for 48 h. Liquid cultures were routinely grown in duplicate in 250-mL Erlenmeyer flasks containing 100 mL of the growth medium. The uninoculated medium was routinely included as a negative control.

### Enrichment of the AK6 mixed culture

Soil samples (2 g) were inoculated into 100 mL of sterilized CDM supplemented with 0.1 mM DBT as a sulfur source and 10 mM of glucose as a carbon source. The enrichment flasks were incubated on a rotary shaker for 4–7 days until turbidity appeared. Subsequently, 1 mL from those original enrichments was transferred to a fresh medium with the same composition and further incubated under the same conditions for the same time. This sub-culturing was repeated 4 times. To check whether AK6 is a pure or mixed culture, samples from enrichment cultures were serially diluted in sterile saline solution (0.9% NaCl) and aliquots from those culture dilutions (100 μL) were spread over LB-agar plates and incubated for 72 h.

### Growth of AK6 on different sulfur sources

The AK6 mixed culture was grown in CDM containing glucose as a carbon source and one of the organosulfur compounds as a sole sulfur source. Another set of cultures was prepared in which the organosulfur compounds served as carbon and sulfur source (no glucose was added). Inocula were prepared from cultures containing the respective sulfur source. AK6 was also grown in CDM containing DBT added as solid without solvent (ethanol) and glucose. To test the ability of AK6 to utilize ethanol as a carbons source, it was cultured in CDM containing ethanol (0.1%) as a sole carbon source and MgSO_4_ as a sole sulfur source. In another experiment, AK6 was grown in CDM containing 2-HBP (0.1 mM dissolved in ethanol) as a carbon source in the presence of MgSO_4_ (1 mM) as a sulfur source. The inoculum for this culture originated from a starter culture grown in the same medium. Growth was monitored by measuring culture turbidity (Optical Density at 600 nm, OD_600_) after time intervals until the culture entered the stationary phase. The biomass yield was measured as dry cell weight (dcw) by drying cell pellets at 105°C for 15 h.

### Biodesulfurization of DBT by cell suspension of AK6

AK6 was grown as described earlier in 1 L Erlenmeyer flasks (duplicates) containing 400 mL of CDM-DBT-glucose and the cells were harvested at the mid-exponential phase (OD_600_ = 0.7) by centrifugation (10,000 rpm for 10 min). The cell pellet was washed twice with phosphate buffer (50 mL, 100 mM, pH 7) and resuspended in 25 mL of the same buffer (2.6 g dcw L^−1^). Both cell suspensions were incubated with 1.0 mM DBT in 100 mL Erlenmeyer flasks at 30°C with shaking (180 rpm) for 4 h. A flask containing phosphate buffer and DBT only without cells was included as a negative control. Samples (1 mL) were retrieved at intervals, and the cells were removed by centrifugation (14,000 rpm for 5 min). Benzotiophene was then added as an internal standard (1 mM). The biodesulfurization intermediates were extracted twice from the cell-free supernatants with one volume of ethylacetate. The organic phase evaporated by centrifugation under vacuum and the residue was resuspended in 200 μL ethanol for HPLC analysis. The produced 2-HBP was quantified from a standard curve by estimating the peak area. The biodesulfurization activity of the AK6 cell suspension was calculated as the amount of 2-HBP produced per g dcw per hour (μM 2-HBP g dcw^−1^ h^−1^).

### Biodesulfurization pathway intermediates

AK6 was grown as described earlier in 2 L Erlenmeyer flasks containing 1000 mL of CDM-DBT-glucose and the cells were harvested in the mid-exponential phase by centrifugation (10,000 rpm for 10 min). The cell pellet was washed with 50 mL phosphate buffer (100 mM, pH 7) and resuspended in 25 mL of the same buffer (6.4 g dcw L^−1^). *R. erythropolis* IGTS8 was grown in CDM-DBT-glucose and treated under the same conditions. The cells of IGTS8 were harvested in the exponential phase after 22 h of incubation, washed and resuspended in 25 mL phosphate buffer (14.8 g dcw L^−1^). Both cell suspensions were incubated with 1.0 mM DBT in 100 mL Erlenmeyer flasks at 30°C with shaking (200 rpm) for 18 h. A flask containing phosphate buffer and DBT only without cells was included as a negative control. All assays were then centrifuged (10,000 rpm for 5 min) to remove the cells. The pH of the supernatants was adjusted to 2 with 25% HCl. Extraction of the intermediates was performed twice by adding one volume (25 mL) of ethylacetate to the acidified supernatants in a separating funnel. After vigorous shaking for 5 min, the mixtures were kept undisturbed to allow phase separation. The aqueous phase was drained, and the organic phase from both extractions was pooled and collected in clean glass flasks. The volume of ethylacetate was reduced by centrifugation under vacuum to 0.5 mL from which samples were analyzed by HPLC and GC/MS

### High-performance liquid chromatography (HPLC)

HPLC was performed on Thermo-Dionex UHPLC 3000 equipped with a photodiode array (Thermo, USA), using an Acclaim™ 120 C18 column (5 μm column, 120 A). Cell-free culture supernatants (500 μL) were extracted once with one volume of ethylacetate that was then evaporated and replaced by 50 μL ethanol. Also, samples from the cell suspension assays were treated in the same way. The mobile phase was an acetonitrile-water mixture (60 and 80%) pumped at a flow rate of 1 mL/min and the injected volume was 10 μL. Detection of the organosulfur compounds and 2-HBP was performed at various wavelengths (233, 235, and 248 nm). Authentic organosulfur compounds and 2-HBP were used for comparison. The concentrations of DBT and 2-HBP were estimated from the peak area using standard curves prepared for both compounds.

### Gas chromatography-mass spectrometry (GC-MS)

The intermediates of DBT biodesulfurization were analyzed with a Gas Chromatograph hyphenated to a mass spectrometer detector (GC-MS) operated in the scanning mode. The system was fitted with a non-polar chromatographic column (DB-1 ht, 30 m long, 0.1 mm ID, and 0.1 μm film thickness). The column was operated under a constant flow of helium as a carrier gas (1.0 mL/min). The GC oven was ramped from 70 to 350°C at 10°C/min. The samples were injected manually into the GC/MS. The collected mass spectra were matched with the NIST mass spectral database.

### Detection of the biodesulfurization genes

Promega Wizard Genomic DNA Purification Kit was used to isolate genomic DNA from cells of the AK6 consortium harvested from CDM cultures with glucose and DBT after 96 h (OD_600_ = 1.2). Primers listed in Table 1 were adopted for the amplification of the *dsz* genes. Genomic DNA from *R. erythropolis* IGTS8 was included as a positive control. The 20-μL PCR assays contained 1 μL (5 ng) of template DNA, 2 μL of each primer (2 pmol/μL), 10 μL Qiagen *Taq* PCR master mix, and 5 μL nuclease-free water. Initial denaturation at 95°C for 2 min was followed by 1 min at 94°C, 1.5 min at 47 or 55°C (annealing), 1 min at 72°C, and eventually a final extension step for 10 min at 72°C.

**Table 1:** **Primers used in this study**.

**Primer name**	**Primer sequence (5′–3′)**	**Target gene**	**Product size (bp)**	**References**
bdsAf	tcgatcagttgtcagggg	*dszA*	547	Davoodi-Dehaghani et al. (2010)
bdsAr	ggatggaccagactgttgac			
bdsBf	atcgaactcgacgtcctcag	*dszB*	422	Davoodi-Dehaghani et al. (2010)
bdsBr	ggaacatcgacaccaggact			
dszCf	acacaccatatgacactgtcacctgaaaaggagc	*dszC*	1250	Kayser et al. (2002)
dszCr	acacacagatcttcaggaggtgaagccgggaatcggg			

### PCR-DGGE analysis of the community structure

#### Extraction of total community DNA

Cell pellets of AK6 were harvested (toward the end of the exponential phase) from CDM cultures containing glucose as a carbon source and different organosulfur compounds. The cell pellets were washed once with 100 mM K-phosphate buffer (pH 7.0) and used as a source for genomic DNA. Total community DNA was extracted with the Ultra Clean Soil DNA purification kit (MoBio Laboratories, USA) according to the manufacturer's instructions. After air drying, DNA pellets were resuspended in 10 μL TE buffer and kept at −20°C until use.

#### Amplification of the 16S rRNA genes by PCR

Amplification of the 16S rRNA genes for DGGE analysis was performed using GC-clamp primers (EUB341F-GC: 5′-CGCC CGCCGCGCGCGGCGGGCGGGGCGGGGGCACGG GGGGCCTACGGGAGGCAGCA GCAG-3′ and EUB517R: 5′-ATTACCGCGGCTGCTGG-3′) which correspond to positions 341 and 517 in *Escherichia coli* genome (Muyzer et al., 1993). Amplifications were performed in 25-μL reactions containing: 2.5 μL of 10x *Taq* buffer (100 mM Tris-HCl, pH 8), 1.25 mM MgCl_2_, 100 μM dNTPs (Invitrogen, USA), 1.2 μM forward and reverse primers (Invitrogen, USA), 0.5 U *Taq* DNA polymerase (Invitrogen, USA), and about 5 ng of template DNA. PCR was performed in Thermal Cycler (Applied Biosystem 2720, USA). A touchdown PCR program was implemented as follows: initial denaturation at 95°C for 5 min; 5 cycles of 94°C for 40 s, annealing at 65°C for 40 s, and extension at 72°C for 40 s; 5 cycles of 94°C for 40 s, annealing at 60°C for 40 s, and extension at 72°C for 40 s; 10 cycles of 94°C for 40 s, annealing at 55°C for 40 s, and extension at 72°C for 40 s; 10 cycles of 94°C for 40 s, annealing at 50°C for 40 s, and extension at 72°C for 40 s, followed by a final hold at 72°C for 7 min. Amplicons were analyzed by electrophoresis in 1% agarose gels with the size markers (1 kb DNA ladder, Invitrogen, USA) and visualized using ethidium bromide.

#### Denaturing gradient gel electrophoresis (DGGE)

DGGE was performed using Dcode Mutation Detection System (Bio-Rad Laboratories Ltd., Hertfordshire, UK). PCR products were electrophoresed in 0.5 × TAE buffer (1 × TAE buffer is 0.04 M Tris base, 0.02 M sodium acetate, and 10 mM EDTA, pH 7.4) on 8% acrylamide gel containing 25–50% denaturating gradient of formamide and urea. DGGE was conducted at 60°C for 5 h at voltage of 200 V. The gel was stained with SYBR Green I Nucleic acid gel stain (Cambrex Bio Science Rockland, USA), photographed and analyzed for DGGE band profile with a UV gel documentation system (Bio-Rad Laboratories Inc., CA, USA).

#### Sequencing and analysis of the DGGE bands

Dominant DGGE bands were cut off with a sterile scalpel and eluted by incubation in 100 μL TE buffer at 100°C for 5 min. The supernatant was used as a template for PCR amplification. Reamplification of 16S rRNA genes from excised DNA fragments was performed using bacterial primers EUB314F without GC clamp and EUB517R. Amplification was verified by electrophoresis on 1% agarose gel. PCR products were purified using PCR-Clean kit (Promega, USA) according to instructions manual. PCR products were directly sequenced using a BigDye terminator cycle sequencing (Sanger et al., 1977) at GenoScreen sequencing facility (Genoscreen, Lille, France). Sequences of the 16S rRNA genes were analyzed using Blast search facility on NCBI (National Center for Biotechnology Information, National Library of Medicine, USA) database (www.ncbi.nlm.nih.gov/BLAST/). Sequences were compared with their closest matches in the GenBank with nucleotide-nucleotide BLAST to obtain the nearest phylogenetic neighbors. Numerical analysis of the DGGE fingerprints was performed using Quantity One 1D software (BioRad). The total number of DGGE bands was used to reflect the richness of AK6 operational taxonomic units (OTUs; Duarte et al., 2009). Bacterial diversity was estimated based on densitometric measurements and Shannon diversity index (*H*′) (Duarte et al., 2009) according to the following equation:

(1) $$\begin{array}{l} H\prime =-\sum P_{i}\;(\ln\;P_{i})\\ \;P_{i}=n_{i}/N_{i} \end{array}$$

P_*i*_ is the relative intensity of DNA band in the fingerprint, *n*_*i*_ is densitometrically measured intensity of individual DNA band, and *N*_*i*_ is the total amount of DNA in the fingerprint. The relative intensity of each band (*Pi*) was used to express the relative frequency of each phylotype (Moreirinha et al., 2011).

The 16S rRNA gene sequences obtained from the AK6 mixed culture were deposited in GenBank under accession numbers LC011106–LC011116.

## Results

### Enrichment of the DBT-desulfurizing mixed culture AK6

The adopted enrichment procedure produced a microbial culture (AK6) that grew in mineral salts medium containing glucose as a carbon source and DBT as a sole sulfur source after repeated subculturing. The AK6 culture appeared yellow to orange in color and was also able to grow (moderate growth) in mineral salts medium containing DBT (dissolved in ethanol) as both a carbon and sulfur source (no glucose). Spread plates of AK6 culture (on glucose and DBT) dilutions revealed several morphologically distinct colonies indicating that AK6 is a mixed culture.

### Biodesulfurization spectrum of AK6

**Figure 2:**
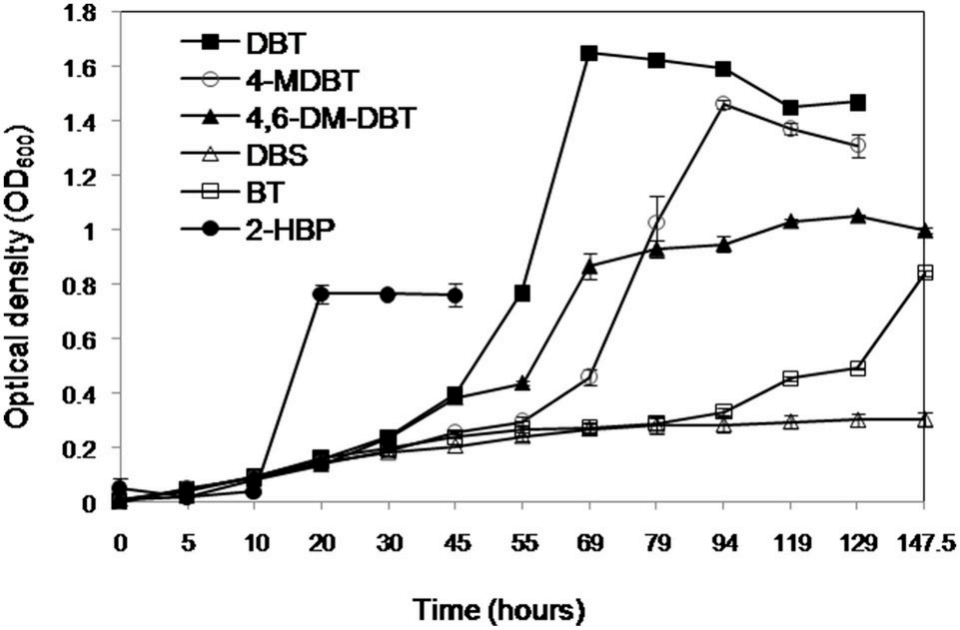
**Growth of the AK6 mixed culture on different organosulfur compounds as sulfur sources in the presence of glucose as a carbon source**. Growth on 2-HBP as a carbon source in the presence of inorganic sulfate as a sulfur source is also shown.

The AK6 culture grew on the organosulfur substrates DBT, BT, 4-MDBT, and 4,6-DM-DBT (all dissolved in ethanol or acetone) as a sole sulfur source in the presence of glucose as a carbon source (Figure 2). DBS (dissolved in ethanol) as a sulfur source supported only residual growth of AK6. HPLC analysis of culture samples revealed the transformation of DBT to a product that co-migrated with authentic 2-HBP. HPLC analysis also clearly showed a decrease in the peaks of BT, 4-MDBT, 4,6-DM-DBT (data not shown). In contrast, no remarkable change occurred in the peak corresponding to DBS. AK6 also gave good growth on 2-HBP (dissolved in ethanol) as a carbon source in the presence of MgSO_4_ as a sole sulfur source (Figure 2). Utilization of 2-HBP by AK6 was confirmed by HPLC (Figure S1).

The AK6 culture gave a moderate growth (maximum OD_600_ = 0.4) in cultures containing DBT as carbon and sulfur source (no glucose). In this case, DBT was added from an ethanol stock solution. In contrast, AK6 did not grow on DBT when it was added to the medium as a solid, without ethanol or any other carbon source. Similarly, AK6 showed reduced growth (maximum OD_600_ = 0.25) when 4-MDBT (from ethanol stock solution) was added to the culture in the absence of any other carbon or sulfur sources. AK6 did not grow on either BT, 4,6-DM-DBT, or DBS as the carbon and sulfur source. Moreover, AK6 grew well in CDM containing ethanol as a sole carbon source and MgSO_4_ as a sulfur source (OD_600_ of 0.8 after 72 h of incubation).

### DBT biodesulfurization activity of the AK6 cell suspension

The biodesulfurization activity of the AK6 mixed culture was estimated as the amount of 2-HBP produced at different time intervals in resting cell assays with DBT as a substrate. After 1 h of incubation, the AK6 cells produced 6.05 ± 0.13 μM 2-HBP g dcw^−1^ h^−1^. This amount increased after 2 h to 9 ± 0.6 μM 2-HBP g dcw^−1^ h^−1^. However, after 4 h of incubation the concentration of 2-HBP declined to 1.9 ± 0.3 μM g dcw^−1^ h^−1^. This indicates that 2-HBP might be consumed with time or, alternatively, transformed into other products.

### DBT biodesulfurization intermediates

**Figure 3:**
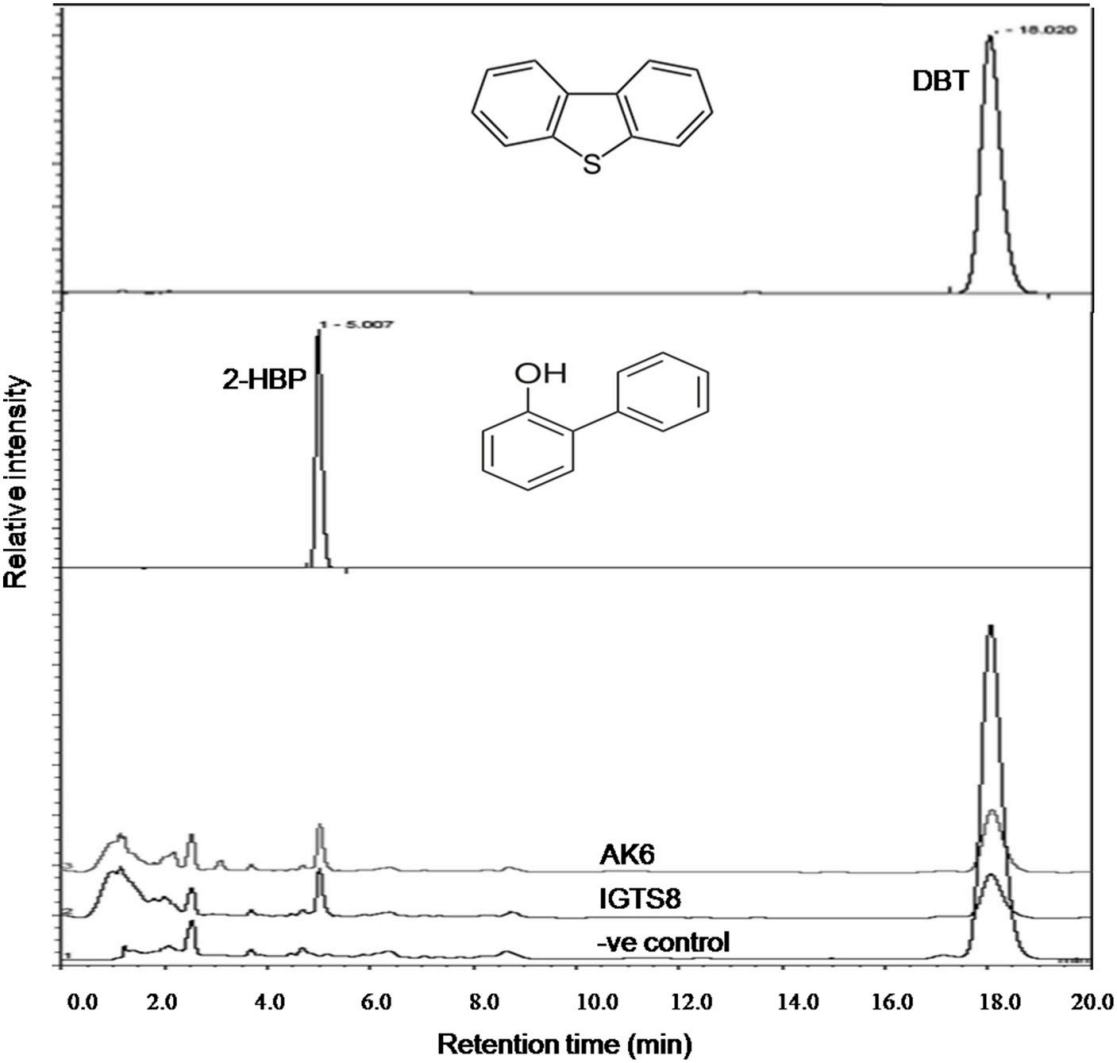
**HPLC chromatogram showing the biotransformation of DBT in resting cell assays for the AK6 mixed culture and the reference strain *R. erythropolis* IGTS8**. The −ve control assay contained buffer and substrate only.

**Figure 4:**
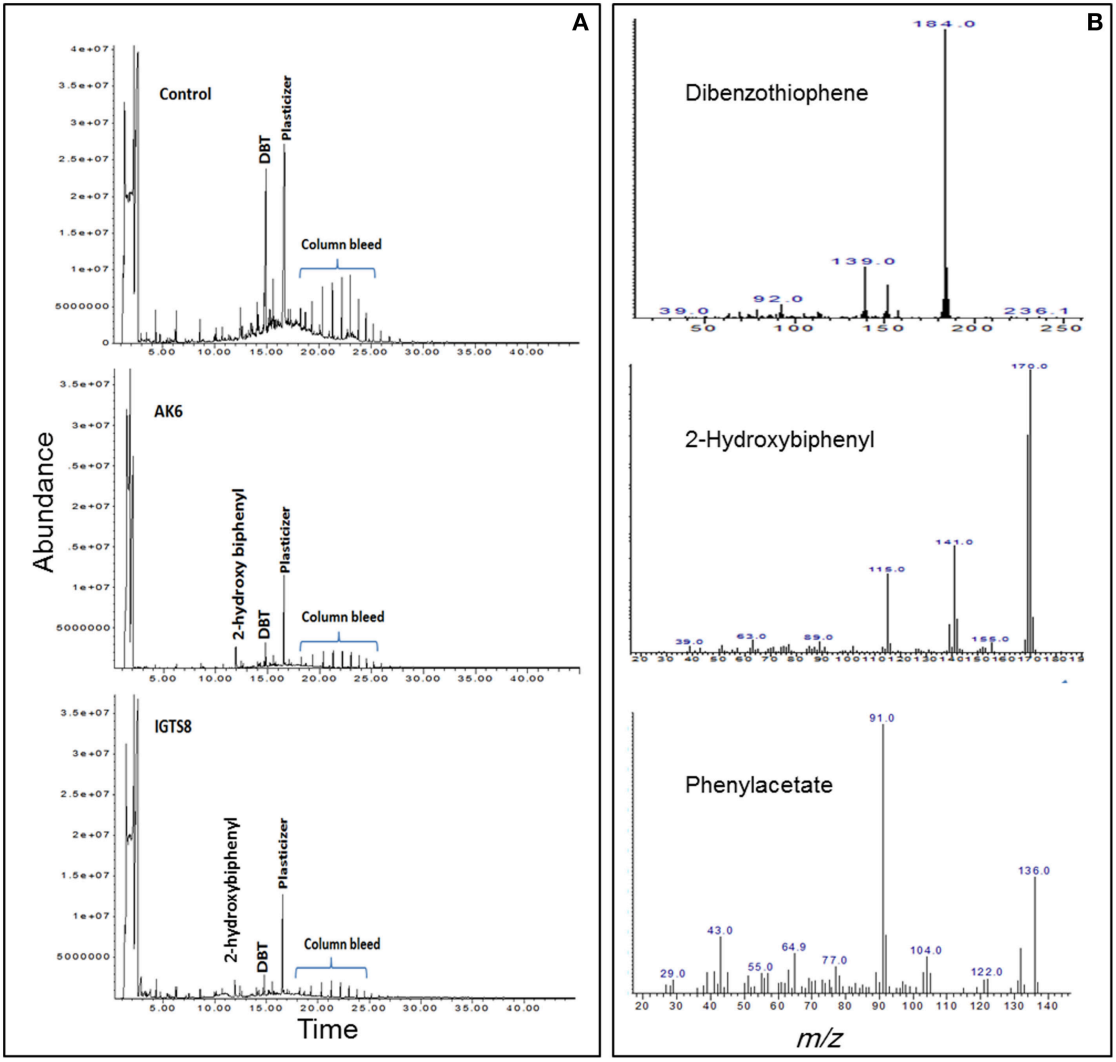
**(A)** Gas chromatogram showing the biotransformation of DBT in resting cell assays for the AK6 mixed culture and the reference strain *R. erythropolis* IGTS8. **(B)** Mass spectra for compounds detected in ethylacetate extracts of the resting cell assays.

HPLC confirmed the biotransformation of DBT by both cell suspensions of AK6 and IGTS8 (positive control). HPLC also revealed a peak comigrating with authentic 2-HBP in both assays (Figure 3). GC-MS analysis revealed several peaks (Figures 4A,B). In all analyzed samples (control, AK6 and IGTS8), two major peaks which were assigned to DBT (at around 15 min, m/z 184) and dibutyl phthalate plasticizer (at 16.5 min, m/z 278) and two minor peaks which were assigned to biphenyl (at 10 min, m/z 154) and benzoic acid (at 8.5 min, m/z 122) were observed. One new major peak (at 12 min, m/z 170) which was assigned to 2-HBP was observed only in AK6 and IGTS8 treatments in addition to the above- mentioned compounds but to less extent. A minor peak (cannot be seen in total ion chromatogram, at around 9 min, m/z 136) which was assigned to phenylacetate was detected only in AK6 assay. Integration of the peak areas for both DBT and 2-HBP revealed that 90% of the added DBT substrate was consumed and nearly 11% of the utilized DBT substrate were recovered as 2-HBP in assays of both AK6 and IGTS8.

### Biodesulfurization genes

**Figure 5:**
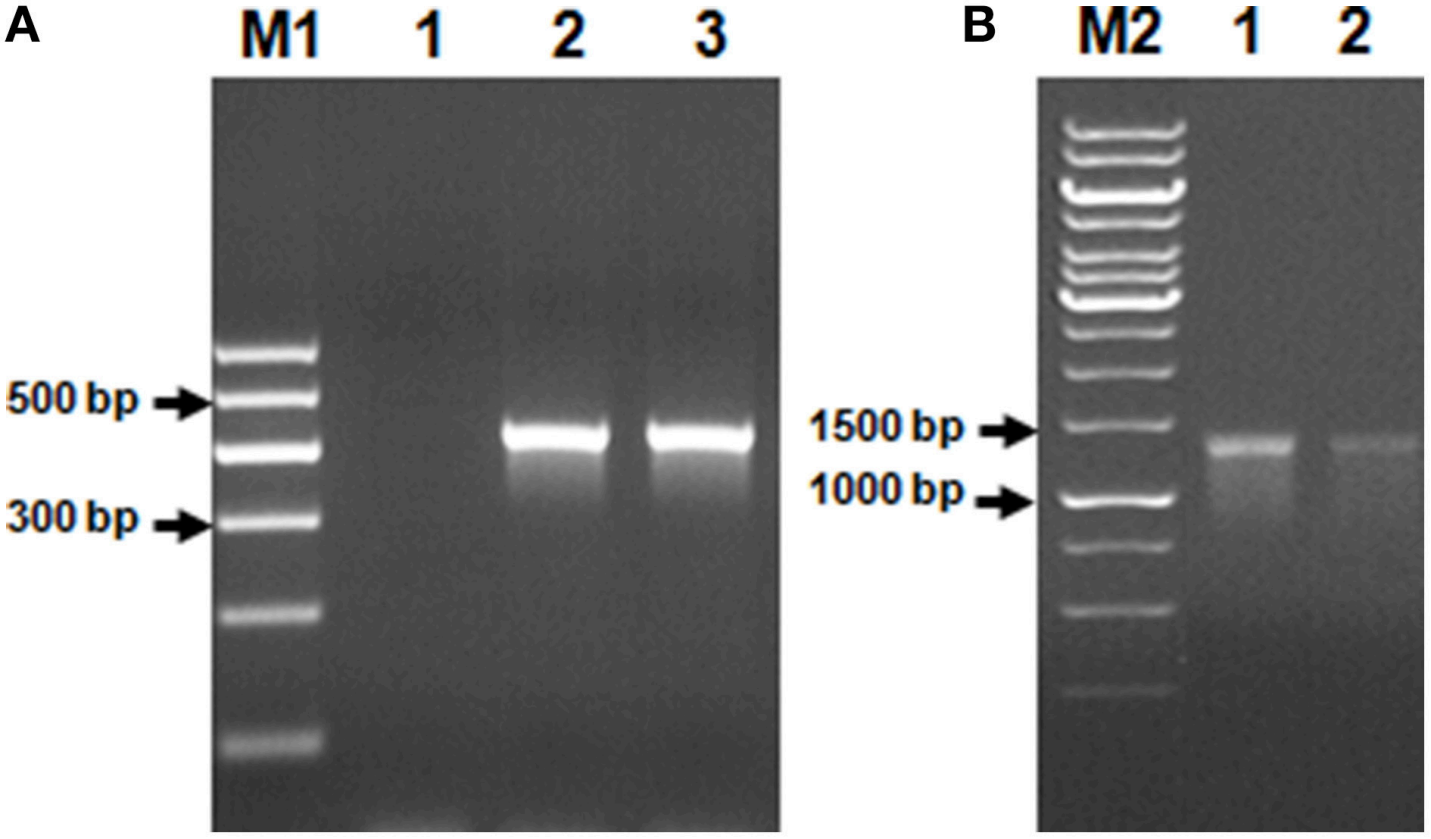
**Genes of the 4S biodesulfurization pathway detected in the AK6 mixed culture**. Genomic DNA from the reference strain *R. erythropolis* was included as a positive control. Lane **A1**: negative (no-template) control, lane **A2**: *dszB* gene in the reference strain, lane **A3**: *dszB* gene in AK6, lane **B1**: *dszC* gene in the reference strain, lane **B2**: *dszC* gene in AK6. M1 and M2 are DNA markers.

Using Genomic DNA isolated from the AK6 community as a template, it was possible to amplify two of the genes commonly found in bacteria possessing the 4S desulfurization pathway, namely, *dszB* and *dszC*. No PCR product corresponding to the *dszA* gene could be obtained (Figure 5).

### Changes in the AK6 community structure (PCR-DGGE)

**Figure 6:**
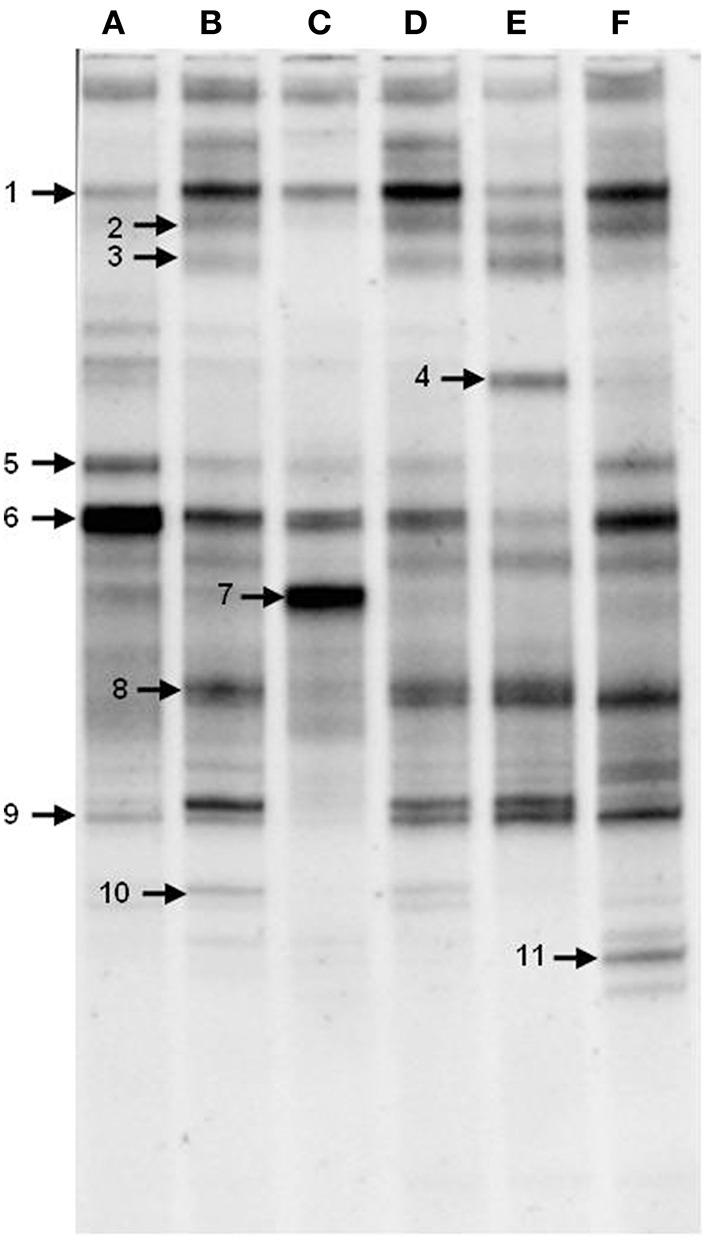
**DGGE profiles of 16S rRNA gene fragments amplified from total community DNA recovered from AK6 cultures grown on different sulfur sources**. Glucose was provided as carbon source for all cultures and dibenzothiophene was also tested without glucose addition. lane **A**, benzothiophene; lane **B** 4,6-dimethyldibenzothiophene; lane **C**, dibenzothiophene (without glucose); lane **D**, dibenzothiophene; lane **E**, dibenzylsulfide; lane **F**, 4-methyldibenzothiophene. Faint DGGE bands representing PCR artifacts were neglected.

To gain insight into the dynamics of the microbial diversity present in AK6 in response to different sulfur sources, bacterial communities in AK6 cultures amended with various organosulfur compounds were monitored using culture-independent PCR-DGGE analysis of 16S rRNA genes. For identification purposes, 16S rRNA genes recovered from dominant DGGE bands were sequenced. The results of homology search and closest matches for the sequences obtained are shown in Table 2. DGGE banding pattern for different cultures reveals a community structure change depending on the provided sulfur source (Figure 6). DGGE profile of cultures supplemented with BT showed the predominance of three major OTUs (operational taxonomic units) identified as members of the genera *Sphingobacterium, Stenotrophomonas*, and *Pseudomonas*. Cultures amended with 4,6-DM-DBT showed more diverse population with at least ten OTUs comprised of *Sphingobacterium, Klebsiella, Pseudomonas*, and *Stenotrophomonas* species. In contrast, the population in cultures grown on DBT in the absence of glucose was restricted to fewer genera assigned to *Sphingobacterium, Stenotrophomonas, Pseudomonas*, and *Cellulosimicrobium*/*Arthrobacter* (Figure 6, Band 7, Table 2), with the latter being more abundant and characteristic to the DBT/no glucose culture. Banding patterns for cultures supplemented with either 4,6-DM-DBT, DBS, or DBT/glucose were similar, consistent and showed almost no change in community structure compared to each other. Dominant DGGE bands from these cultures were affiliated to the genera *Sphingobacterium, Klebsiella, Pseudomonas, Stenotrophomonas*, and uncultured members of *Stenotrophomonas*. The 4-MDBT culture had the largest number of DGGE bands. However, *Klebsiella* spp.-related sequences were lacking. One characteristic feature of the 4-MDBT culture is the presence of *Mycobacterium*/*Rhodococcus* spp. (Figure 6, Band 11, Table 2) along with other dominant bacteria.

**Table 2:** **Bacterial species identified in AK6 cultures and their phylogenetic affiliations**.

**DGGE Bands**	**Accession No**.	**Closest Matches**	**Similarity (%)**	**Accession No**.	**Phylogenetic Affiliation**
A1	LC011106	*Sphingobacterium* sp. S2842	100	KJ939323	Bacteroidetes/Sphingobacteriaceae
		Uncultured *Sphingobacterium* sp. clone OTU0876	100	KM059710	Bacteroidetes/Sphingobacteriaceae
		*Sphingobacterium siyangense* ALS-4	100	KJ638991	Bacteroidetes/Sphingobacteriaceae
		*Sphingobacterium multivorum* M-A-02/11-10-1	100	KF777399	Bacteroidetes/Sphingobacteriaceae
B2	LC011109	*Klebsiella pneumoniae* R18	100	KM017982	Proteobacteria/Enterobacteriaceae
		Uncultured *Klebsiella* sp. clone TSK	100	KF649832	Proteobacteria/Enterobacteriaceae
		*Erwinia chrysanthemi* DSM 4610T	100	HG515379	Proteobacteria/Enterobacteriaceae
		*Klebsiella* sp. XMR21	100	KM241871	Proteobacteria/Enterobacteriaceae
B3	LC011110	*Klebsiella pneumoniae* QLR-8	99	KM096437	Proteobacteria/Enterobacteriaceae
		*Klebsiella variicola* R39	99	KM019912	Proteobacteria/Enterobacteriaceae
		*Klebsiella* sp. M.pstv.26.2	99	KM108517	Proteobacteria/Enterobacteriaceae
		*Klebsiella* sp. F018	99	KJ846494	Proteobacteria/Enterobacteriaceae
E4	LC011115	*Pseudomonas* sp. ESBL485B15-13-4E	100	KJ831548	Proteobacteria/Pseudomonadaceae
		*Pseudomonas plecoglossicida* RS 1	100	KJ508408	Proteobacteria/Pseudomonadaceae
		*Pseudomonas* sp. FSBRY17	100	KJ200400	Proteobacteria/Pseudomonadaceae
		*Pseudomonas putida* FUM1A3	100	KC195910	Proteobacteria/Pseudomonadaceae
A5	LC011107	*Stenotrophomonas maltophilia* B8R	99	DQ466570	Proteobacteria/Xanthomonadaceae
		*Stenotrophomonas* sp. SO5.1	99	KC859435	Proteobacteria/Xanthomonadaceae
		*Stenotrophomonas* sp. CDRIG20	99	JN574752	Proteobacteria/Xanthomonadaceae
		*Xanthomonas* sp. JAPE1	99	KF952249	Proteobacteria/Xanthomonadaceae
A6	LC011108	*Pseudomonas* sp. NTN153	99	LK936599	Proteobacteria/Pseudomonadaceae
		*Pseudomonas* sp. SCU-B128	99	KJ000799	Proteobacteria/Pseudomonadaceae
		*Pseudomonas* sp. SF90	99	JX134078	Proteobacteria/Pseudomonadaceae
		*Pseudomonas* sp. X10	99	EU285592	Proteobacteria/Pseudomonadaceae
C7	LC011114	*Cellulosimicrobium* sp. L414	100	KJ944168	Actinobacteria/Promicromonosporaceae
		Uncultured *Arthrobacter* sp. clone R48	100	KC922044	Actinobacteria/Micrococcaceae
		*Cellulomonas hominis* PuiC5.18	100	LM994741	Actinobacteria/Cellulomonadaceae
		*Cellulosimicrobium cellulans* S17	100	KJ947163	Actinobacteria/Promicromonosporaceae
B8	LC011111	*Pseudomonas putida* NBFPASM-RAS176	99	KJ917221	Proteobacteria/Pseudomonadaceae
		Uncultured *Pseudomonas* sp. clone SDn2-35	99	JX493326	Proteobacteria/Pseudomonadaceae
		*Pseudomonas azotifigens* 6H33b	99	NR041247	Proteobacteria/Pseudomonadaceae
		Uncultured *Proteobacterium* clone Upland-16-5526	99	JF986228	Proteobacteria/Environmental sample
B9	LC011112	Uncultured *Stenotrophomonas* sp. clone DVASW-J329	99	KF722572	Proteobacteria/Xanthomonadaceae
		Uncultured *Stenotrophomonas* sp. clone WCD37	99	KJ123780	Proteobacteria/Xanthomonadaceae
		Uncultured *Stenotrophomonas* sp. clone DVBSD-J259	99	KF463873	Proteobacteria/Xanthomonadaceae
		Uncultured bacterium clone MW75	99	JN868813	Bacteria/Environmental sample
B10	LC011113	*Pseudomonas hibiscicola* R8-737	99	JQ659977	Proteobacteria/Pseudomonadaceae
		*Stenotrophomonas maltophilia* TCCC11385	99	FJ393320	Proteobacteria/Xanthomonadaceae
		*Stenotrophomonas* sp. F802	99	AY371433	Proteobacteria/Xanthomonadaceae
		*Pseudomonas* sp. NTN153	99	LK936599	Proteobacteria/Pseudomonadaceae
F11	LC011116	*Mycobacterium* sp. SR34	100	KF896115	Actinobacteria/Mycobacteriaceae
		*Mycobacterium* sp. RDB-148	100	AB730319	Actinobacteria/Mycobacteriaceae
		*Rhodococcus* sp. ZWL3NT	100	JX512559	Actinobacteria/Nocardiaceae
		*Rhodococcus* sp. NCCP-309	100	AB734810	Actinobacteria/Nocardiaceae

**Figure 7:**
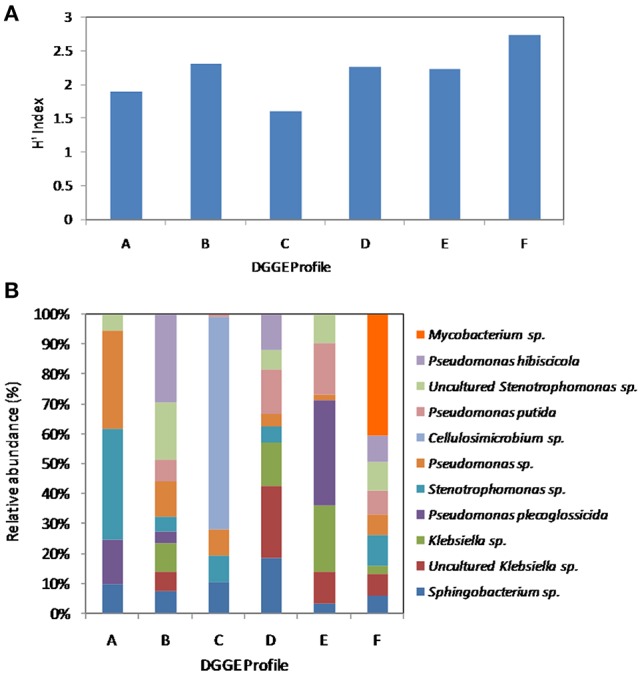
**Numerical analysis of the DGGE fingerprints revealing bacterial diversity among AK6 cultures grown on different sulfur sources**. **(A)** Sulfur source-dependent change in the diversity index (H′). **(B)** Species richness and abundance of various bacterial genera across the different AK6 bacterial communities. Glucose was provided as carbon source for all cultures and dibenzothiophene was also tested without glucose addition. A, benzothiophene; B 4,6-dimethyldibenzothiophene; C, dibenzothiophene (without glucose); D, dibenzothiophene; E, dibenzylsulfide; F, 4-methyldibenzothiophene.

Estimation of bacterial diversity was possible using numerical analysis of the DGGE fingerprints. Overall bacterial richness exhibited different trends with different sulfur sources. The bacterial community enriched on 4-MDBT had the highest operational taxonomic OTU richness. The lowest richness was observed in the DBT/no glucose cultures. OTU richness correlated positively with bacterial diversity. Diversity as measured by H′ index, was variable depending on the sulfur source (Figure 7A). Cultures grown on 4-MDBT had relatively high species diversity followed by those grown on BT. On the other hand, cultures grown on DBT without glucose had relatively low species diversity. Besides diversity, species abundance also varied depending on the utilized sulfur source (Figure 7B). Species abundance provided complementary insights regarding the effect of the provided sulfur source on bacterial community structure. Specifically, the most abundant OTUs across all cultures were members of the genera *Sphingobacterium* and *Pseudomonas*. Some OTUs appeared to be sulfur source-specific. In this regard, *Mycobacterium/Rhodococcus* sp. and *Cellulosimicrobium*/*Arthrobacter* sp. were restricted to the 4-MDBT and DBT/no glucose cultures, respectively.

## Discussion

We selected some thiophene compounds to investigate the biodesulfurization potential of the microbial mixed culture AK6. These compounds were selected because they constitute the major fraction of organosulfur in crude oil and diesel. Thiophenic compounds account for about 70% of the sulfur contained in crude oil (Borgne and Quintero, 2003). Furthermore, they are resistant to the conventional hydrodesulfurization, and it is important to remove them to drastically reduce the sulfur content of diesel as mandated by environmental regulations (Ohshiro and Izumi, 1999). The adopted enrichment procedure produced a microbial culture that utilized DBT as a sole sulfur source in the presence of glucose as a carbon source. In agreement with these results, many authors reported the isolation of biodesulfurization-competent microorganisms from soil contaminated with hydrocarbons or crude oil (Monot and Warzywoda, 2008; Mohamed et al., 2015). The observation of several morphologically distinct colonies on spread plates confirmed that AK6 is a mixed culture. Deployment of mixed cultures and engineered consortia in biodesulfurization research, though very rare, has been reported by some authors. Li and Jiang (2013) and Jiang et al. (2014) studied biodesulfurization of model thiophenic compounds and heavy oil by mixed cultures enriched from oil sludge. Most recently, Martínez et al. (2016) reported enhanced desulfurization of oil sulfur compounds by using engineered synthetic bacterial consortia.

Growth of the AK6 culture on several organosulfur compounds in the presence of glucose and HPLC analysis confirmed the ability of AK6 to utilize those substrates as sulfur sources, except DBS. Moreover, lack of growth on the tested organosulfur compounds in the absence of glucose suggests that AK6 can't utilize them as a carbon source. The moderate growth observed on DBT (as a carbon and sulfur source) is most likely due to the utilization of ethanol in which DBT was dissolved. The capability of different bacteria to utilize ethanol as an efficient carbon source for enhanced desulfurization of DBT has been reported (Aggarwal et al., 2013). In line with this, AK6 grew on ethanol as a sole carbon source in the presence of MgSO_4_ as a sulfur source. Furthermore, AK6 could not grow when DBT was added as solid to the growth medium (no glucose, no ethanol). Altogether, the growth experiments and HPLC analysis confirmed the joint capability of the bacterial assortment present in AK6 to utilize a broad spectrum of organosulfur substrates (DBT, BT, 4-MDBT, 4,6-DM-DBT) only as a sulfur source.

The genetic determinants of the 4S pathway have been the target for enormous genetic boosting strategies. Nevertheless, none of the genetically improved axenic cultures has shown biodesulfurization rate meeting the industrial requirements for the development of a commercial biodesulfurization process (Kilbane, 2006; Boniek et al., 2015; Mohamed et al., 2015). Interestingly, other host factors which are not linked with the genetics of the 4S pathway have shown synergetic effect enhancing the biodesulfurization rate (Kilbane, 2006). Accordingly, a consortium of biodesulfurization-competent strains such as AK6 would be a rich pool of these host factors for more efficient and robust biodesulfurization process. The AK6 mixed culture seems to contain both desulfurizing as well as non-desulfurizing bacteria which are adapted to co-metabolize the 4S pathway intermediates. This mosaic nature of AK6 is expected to fit better than axenic monocultures for the development of biotechnological processes targeting desulfurization of a broad range of sulfur compounds present in crude oil and diesel. Furthermore, deployment of mixed cultures may avoid the decay of the biodesulfurization activity resulting from the accumulation of the inhibitory intermediates of the 4S pathway (Abin-Fuentes et al., 2013; Martínez et al., 2016). It remains, however, to identify which members of the AK6 community are essential for the utilization of each of the tested organosulfur compounds. In other words, it is interesting to investigate if the type of the sulfur source has an impact on the structure of the AK6 community. One approach to address these issues would be the time-dependent isolation of the bacterial strains from each AK6 culture. Then, each isolate should be investigated individually and in different qualitative and quantitative combinations with others regarding substrate spectrum, biodesulfurization efficiency, and mechanism. The latter should cover the pathway intermediates and the genetic background. Obviously, this culture-dependent approach is laborious and time-consuming (van Hamme et al., 2003). Due to the inherent bias and limited recovery efficiency of the culture-dependent approach, it is probable that we miss one or more of the community members essential for the utilization of a particular sulfur source. Furthermore, isolation of a microorganism in a pure culture does not necessarily reveal its role in the community.

Culture-independent molecular characterization techniques have been shown to be more useful for analyzing microbial components of consortia responding actively in desulfurization of thiophene substrates (Duarte et al., 2001). Direct amplification of the *dsz* genes, which code for the biodesulfurization enzymes via the 4S pathway, or testing their expression rates by RT-qPCR would not be discriminative for the identification of the active bacterial groups in AK6 because of the high sequence similarity of the *dsz* genes among different bacterial groups. Alternatively, 16S rRNA gene-based qPCR for the identification and quantification of biodesulfurization-active bacterial components in the AK6 culture would be applicable. However, this could be misleading because other non-biodesulfurizing bacteria in AK6 which can utilize and grow on the intermediates of the 4S pathway will also be detected. Therefore, we chose to perform the investigations using the PCR-based DGGE analysis of 16S rRNA gene fragments amplified directly from total community DNA recovered from the mixed culture. This culture-independent technique excludes the bias associated with the culture media, provides identification of microorganisms by sequencing the gel bands, and gives an overview of bulk changes in community structure (van Hamme et al., 2003). Analysis of AK6 community dynamics revealed qualitative as well as quantitative changes depending on the utilized sulfur source. This variation reflects the metabolic specialization of the bacterial components of AK6. As one might expect, the dominant strains in each culture are those having the ability to desulfurize or (co)metabolize the respective organosulfur compound. In fact, all detected 16S rRNA gene sequences are related to those of bacterial genera that are known to be biodesulfurization-competent, hydrocarbon degraders, or inhabitants of hydrocarbons-polluted environments (Duarte et al., 2001; Mohebali and Ball, 2008; Bhatia and Sharma, 2012; Ismail et al., 2014). The presence, in the majority of the cultures, of *Pseudomonas, Sphingobacterium, Klebsiella*, and *Stenotrophomonas* spp.-related sequences indicates the significant role of these bacteria in the biodesulfurization of different organosulfur substrates. This can be reconciled for *Pseudomonas, Klebsiella*, and *Stenotrophomonas* spp., which have been reported in several biodesulfurization studies. In contrast, there are no reports in the literature concerning biodesulfurization by *Sphingobacterium* spp. However, these bacteria have been implicated in the biodegradation of polycyclic aromatic hydrocarbons (PAH) and lubricating oil in addition to biosurfactants production (Kanaly et al., 2000; Noparat et al., 2014). Accordingly, *Sphingobacterium* spp. might play an indirect role in the utilization of the organosulfur substrates by providing the community with essential nutrients and surfactants or detoxification of toxic degradation intermediates (Sathishkumar et al., 2008; McGenity et al., 2012; Mikesková et al., 2012; Todorova et al., 2014). Nonetheless, a direct role in the biodesulfurization process can't be ruled out

The 16S rRNA gene sequences retrieved from DGGE band number 7, which existed exclusively in AK6 cultures supplemented with DBT only (dissolved in ethanol) as the sole carbon and sulfur source, were identical to Actinobacterial genera like *Cellulosimicrobium* and *Arthrobacter*. It can be proposed that these bacteria play a significant role in DBT metabolism. The observed moderate growth in this culture was supported most probably by the solvent ethanol as a carbon source and DBT as a sulfur source. Some *Arthrobacter* spp. can utilize DBT and its alkylated derivatives as a sulfur source (Lee et al., 1995; Duarte et al., 2001). Accordingly, *Arthrobacter* spp. are better candidates for the DGGE band 7 than *Cellulosimicrobium* spp. which have not been reported as biodesulfurization-competent. However, it can't be excluded that *Cellulosimicrobium* spp. identified in band 7 are not involved in the biodesulfurization process. Instead, they might have grown on the biodesulfurization intermediates or fed on the debris of dead cells, particularly sugar components (Vogt et al., 2005).

The DGGE band 11 was detected only in the 4-MDBT culture. Therefore, the underlying organisms appear to be involved in the utilization of 4-MDBT as a sulfur source. This is consistent with many studies that reported the biodesulfurization capabilities of some *Mycobacterium* and *Rhodococcus* spp. (Mohebali and Ball, 2008). The DGGE bands detected in the DBS/glucose cultures represent bacterial components of AK6 that gave the residual growth in those cultures. This residual growth was probably supported by glucose and sulfur traces in the growth medium.

The DGGE data provided interesting insights into the effect of the sulfur source on the structure of the AK6 community and the bacterial members that are probably the major contributors. It is worth noting that for the DGGE analysis we focused only on the major bands in the DGGE gels. Therefore, we can't exclude the presence of other bands, minor or not detectable on the gels; that represent other contributors to the biodesulfurization process, which might be directly or indirectly involved. In this context, it has been also reported that a single DGGE band might represent multiple strains (Sekiguchi et al., 2001; Al-Awadhi et al., 2013). Furthermore, the PCR-associated limitations due to primer specificity and differential/preferential amplification of 16S rRNA genes might lead to bias in the structure of the microbial community (Polz and Cavanaugh, 1998; Sipos et al., 2007). This obviates the need for deeper and more conclusive analysis through a combination of approaches to circumvent the limitations inherent to each approach. Our current study on the AK6 mixed culture opened several interesting questions that represent the basis for further in-depth investigations such as: How many bacterial strains exist in each AK6 culture? Which components are indispensable for the biodesulfurization process? What is the role of each AK6 member in the biodesulfurization process? How the AK6 community behaves in the presence of a mixture of organosulfur sources? Can the AK6 community survive in biphasic media? Can the AK6 mixed culture remove sulfur from diesel and gasoline? How active and efficient is AK6 in biodesulfurization as compared to axenic cultures? How reproducible is the AK6 community? All these questions need to be addressed by applying metagenomics and deeper sequencing via next generation techniques. Metagenomic investigations by sequencing and analysis of the 16S rRNA gene pool should enable comprehensive fingerprinting of the AK6 community under different culturing conditions. Alternatively, direct shotgun sequencing on the metagenome shall allow *de novo* assembly of the microbial community as well as compositional analysis in terms of the functional genes. This, in addition to systems biology approaches like metatranscriptomics and metaproteomics coupled to the use of isotope-labeled substrates and biochemical analysis, should provide essential information that will allow the development and engineering of microbial consortia for efficient and economically viable biorefining processes for the fossil fuel industry. In this context, Martínez et al. (2016) have reported a novel approach utilizing engineered synthetic bacterial consortia for enhanced desulfurization and revalorization of oil sulfur compounds. This new approach was developed to overcome inhibition of the Dsz enzymes by the 4S pathway intermediates, and to enable efficient production of value-added intermediates, e.g., 2-(2′-hydroxyphenyl) benzene sulfinate (HBPS), that are difficult to obtain with monocultures.

The detection of 2-HBP, a characteristic end product of the 4S pathway, in the AK6 resting cell assays confirms that DBT biodesulfurization follows the 4S pathway. This is consistent with the detection of *dszB* and *dszC* genes. The lack of a *dszA* PCR product may be due to an insufficient specificity of the used primers. Alternatively, DszA might be lacking in the AK6 community, and another enzyme compensates its catalytic role in the 4S pathway. These findings together with the detection of sequences related to *Rhodococcus, Mycobacterium, Arthrobacter, Stenotrophomonas*, and *Klebsiella* spp., known to harbor the 4S pathway, provide a solid evidence that these bacteria are key players in the biodesulfurizing cultures. It can't be, however, excluded that other hydrocarbons biodegradation/biotransformation pathways are involved. The actual involvement of the 4S and other pathways in the AK6-mediated biodesulfurization process should be further investigated by monitoring temporal changes in gene expression via RT-qPCR.

Previous studies on biodesulfurization of DBT reported specific activities higher and lower than those reported here for AK6 resting cells. These differences could not be attributed solely to the microbial cultures tested in the different studies. The culture conditions, assay design, and analytical approach should also be considered (Kilbane, 2006; Mohamed et al., 2015). The amount of 2-HBP recovered in the cell suspension assays was tiny and decreased with time. Some authors reported that 2-HBP production was not stoichiometric with the amount of transformed DBT (Davoodi-Dehaghani et al., 2010; Mohamed et al., 2015). The AK6 resting cells originate from DBT-glucose cultures, which include the majority of the bacterial groups detected in AK6 consortium. Accordingly, further transformation of DBT or 2-HBP into other aromatic products can't be excluded. In line with this, the ability of AK6 to grow on 2-HBP as a carbon source might lead to biodegradation/biotransformation of 2-HBP to more polar products. The samples were further analyzed by full-scan mode GC/MS to test this possibility. Dibutylphthalate, a well-known plasticizer, was detected in control samples lacking AK6 and to a lesser extent in AK6 and IGTS8. Therefore, the biotic formation of this compound as a product of DBT or 2-HBP transformations by AK6 was excluded.

Benzoate, a confirmed intermediate in the degradation of either DBT via the angular dioxygenase pathway (Nojiri et al., 2001) or 2-HBP which is encoded by the *hbp* genes (García et al., 2014) was detected to a lower extent in AK6 compared to the control. None of the preceding intermediates that lead to the formation of benzoate in these pathways could be detected. Besides, benzoate was also detected in the cell-free control assays. Accordingly, the biotic generation of benzoate from DBT or 2-HBP by AK6 was also excluded. Similarly, the formation of biphenyl by AK6, a possible intermediate in an extended pathway for desulfurization of DBT (Akhtar et al., 2009), was excluded since it was detected mainly in control samples and to lower extent in AK6 assay. Although the transformation profiles of DBT by AK6 consortium and the reference IGTS8 strain were similar (Figure 4), a minor peak assigned to phenylacetate was detected only in AK6 assay. Pure cultures have not been reported to produce phenylacetate as an intermediate in the degradation pathways of DBT or 2-HBP. However, since AK6 is a consortium containing a variety of hydrocarbon-degrading bacteria, the biotic formation of phenylacetate can't be ruled out. Since AK6 is a mixed culture and 2-HBP is a toxic phenolic biocide that can damage cell membranes, we can speculate that phenylacetate originates from 2-HBP via an unknown detoxification mechanism by one or more of the AK6 bacterial components. However, the likelihood of 2-HBP conversion to phenylacetate by AK6 bacteria lacking the biodesulfurization activity can't be ruled out. In this case, phenylacetate is not considered as a new intermediate in an extended 4S pathway, rather it is a product of cometabolism of a sulfur-free substrate (2-HBP).

The proposed transformation of 2-HBP to phenylacetate could probably enhance the biodesulfurization activity of AK6 by eliminating the toxic and inhibitory effect of 2-HBP. This is further corroborated by the ability of AK6 to grow on 2-HBP as a carbon source.

Application of mixed microbial cultures for biodegradation and biotransformation of hydrocarbons is perceived as advantageous. This is due to interspecific and intraspecific interactions that enable microbial consortia to out-perform pure cultures (McGenity et al., 2012; Mikesková et al., 2012; Seth and Taga, 2014). Kim et al. (2009) reported a higher efficiency of phenanthrene degradation by a microbial consortium as compared to the degradation efficiency of the individual component strains. Although the microbial interactions in mixed populations or consortia are not well understood, some types of interactions have been reported in the literature such as collaborative degradation or transformation of the substrate, removal, or sequestration of toxic intermediates, provision of essential metabolites and coenzymes (Konopka et al., 2015).

To summarize, it seems that the major peak of dibutylphthalate and the minor peaks of biphenyl and benzoate detected in the assays most likely are contaminants, and the possibility of the biotic formation of phenylacetate needs further investigation.

## Conclusions

A mixed bacterial culture AK6 was enriched from hydrocarbons-polluted soil based on its biodesulfurization competency. The type of the utilized organosulfur source had an impact on the structure of the AK6 community. The AK6 culture showed a good biodesulfurization substrate spectrum and higher DBT biodesulfurization efficiency. Biodesulfurization of DBT proceeds via the non-destructive 4S pathway and, probably other pathways. Mixed cultures hold a promising potential for the development of biocatalytic desulfurization technology and deserve further in-depth investigations.

## Author contributions

WI designed research, analyzed and interpreted data, and wrote the manuscript. WE and MM conducted experiments and wrote the manuscript. AA and AE conducted experiments.

### Conflict of interest statement

The authors declare that the research was conducted in the absence of any commercial or financial relationships that could be construed as a potential conflict of interest.

## Supplementary material

Figure S1 **HPLC analysis of 2-HBP utilization by AK6 growing in mineral salts medium with 2-HBP as a carbon source and MgSO_4_ as a sole sulfur source**.
