# Supplementary figures and images for: Biocatalytic Desulfurization Capabilities of a Mixed Culture during Non-Destructive Utilization of Recalcitrant Organosulfur Compounds
Source: Front Microbiol. 2016 Mar 3;7:266. doi: 10.3389/fmicb.2016.00266 (PMC4776162; doi:10.3389/fmicb.2016.00266)

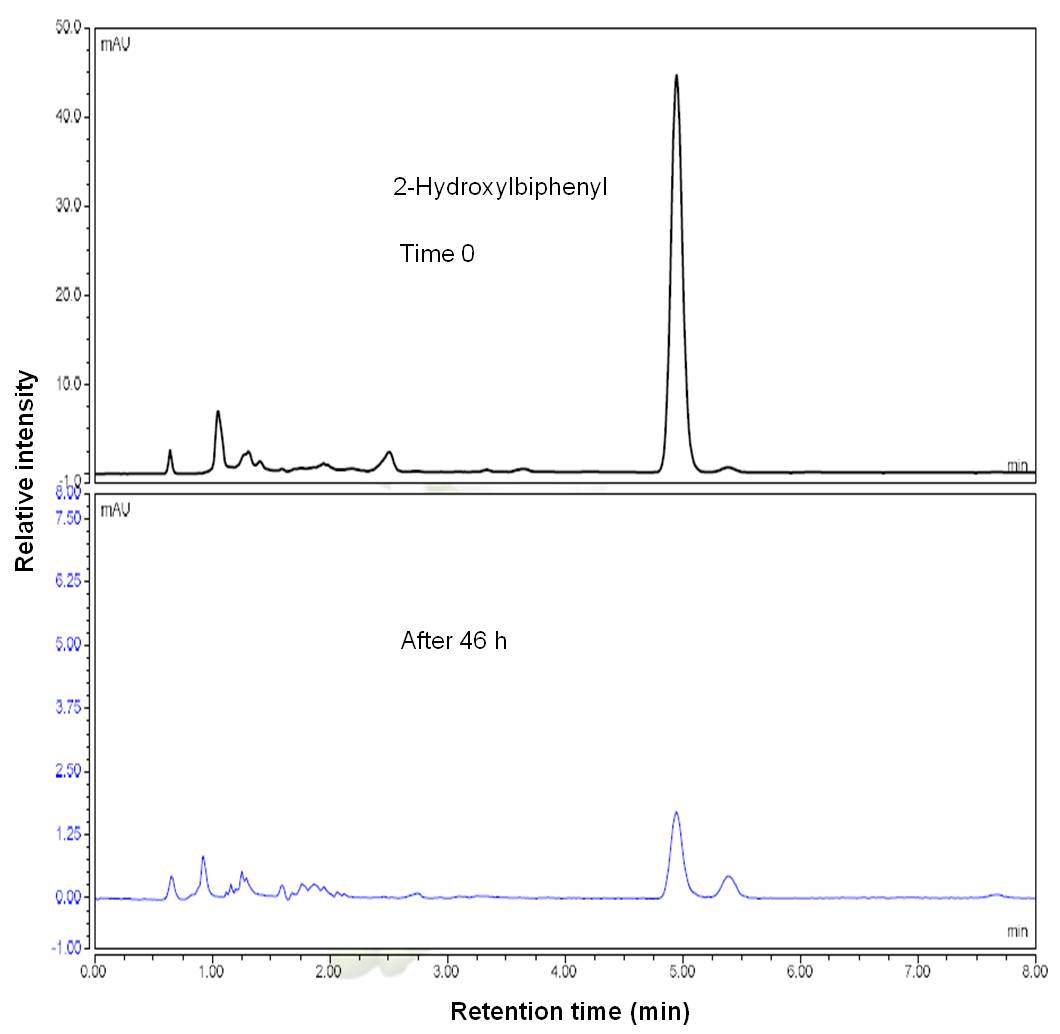

Supplement: Figure S1 — HPLC analysis of 2-HBP utilization by AK6 growing in mineral salts medium with 2-HBP as a carbon source and MgSO4 as a sole sulfur source. [file Image1.JPEG]
